# Banquet or No Banquet

**Published:** 1887-03

**Authors:** 


### BANQUET OR NO BANQUET.

For many years, perhaps ever since the organization of the State Medical Association, it has been the custom in Texas to entertain the delegates to the State Medical Convention at a banquet—usually on the last evening of the session. Of recent years, however, there has appeared opposition to the custom on the part of many members; and in '84, when the State Medical Association met in Belton, an exception was made. At Houston the next year, a resolution opposing the custom, was only defeated out of courtesy to the committee of arrangement, whose invitation to a banquet had just been accepted. At Dallas a magnificent spread was given and much enjoyed. Now just on the eve of the meeting of the State Association at the Capital—for the first time since '75, and the committee of arrangements consisting of the entire local profession, are considering ways and means for the entertainment of the distinguished guests, the question becomes one of great moment, especially in view of the temperance agitation.

A banquet, without wine, would be a hollow mockery. The action taken on this occasion will be significant, and not without influence for good or evil. Five hundred picked delegates, presumably representing five thousand Texas physicians—the entire profession of the State—by their endorsement of a wine banquet throw the moral weight of the medical profession against the great cause of temperance, which has in other States, Kansas, notably, accomplished so much good. By abolishing the custom—and the medical profession of the capital of the great State of Texas taking the initiative—the moral influence of the medical profession is tacitly thrown in the scale of temperance. At every meeting of the State Association recently, petitions have been read from the Womens' Christian Temperance Union, asking the co-operation of the medical profession in putting down the use of liquor, and individually, many members are warmly seconding their work.

We do not propose to preach a temperance sermon, but the JOURNAL here and now desires to be put on record as setting its face firmly against the custom of entertaining the medical delegates with wine; and, while it is argued that more will be expected in the way of entertainment at the capital than at the smaller towns, and that it would look shabby in Austin to make an innovation, it can be answered that as elegant, and at least less questionable, entertainment can be provided, in some other way, and we hope the committee of arrangements will have the moral courage to set the example of dispensing with the use of wine, and thus, it is hoped, forever break up the custom. It may be objected to by some; but we venture the majority of the more thoughtful members will heartily endorse it and applaud Austin for the move; ultimately it will be cause for rejoicing. It would have a deeper significance and a more far reaching influence than would at first appear. It would amount to an emphatic expression on the subject, from a large and enlightened class of people—an endorsement of the effort being made to suppress the evil of intemperance. It is claimed by the prohibition speakers—and the figures may be exaggerated, that nineteen million dollars annually are spent for liquor in Texas! This is more than double the amount spent for bread; and there is no wonder that our people complain of hard times and scarcity of money when such an incubus is on them. Were it removed, as it must and will be some day, reaction will follow like that of a rubber ball on the rebound, and prosperity, instead of paralysis, will characterize every industry of the State.

